# A cross‐lagged panel model examining the longitudinal associations between maternal emotion regulation difficulties, parenting stress, and child socio‐emotional problems in toddlerhood

**DOI:** 10.1002/imhj.70051

**Published:** 2025-10-06

**Authors:** Charlotte Verhagen, Stefanie Duijndam, Nina Kupper, Paul Lodder, Myrthe Boekhorst

**Affiliations:** ^1^ Department of Medical and Clinical Psychology, CoRPS – Center of Research on Psychological Disorders and Somatic Diseases Tilburg University Tilburg the Netherlands; ^2^ Department of Methodology Tilburg University Tilburg the Netherlands

**Keywords:** child socio‐emotional problems, longitudinal, parent emotion regulation difficulties, parenting stress, toddlerhood

## Abstract

In toddlerhood, parents play a crucial role in supporting the socio‐emotional development of children through their parenting behaviors. Certain parental risk factors, such as emotion regulation difficulties and parenting stress, have been found to be related to both parenting practices and child outcomes. As the interplay between these risk factors and their associations with child socio‐emotional problems can change across toddlerhood, the aim of this study was to investigate the longitudinal, bidirectional associations of parenting stress and maternal emotion regulation difficulties with child socio‐emotional problems. This study included 360 mothers from the Southern regions of the Netherlands (*M*_age_ = 31.4, 90.7% Dutch, 81.4% completed university) who completed questionnaires biannually (Jan 2021–May 2024) within the first 3 years postpartum, as part of a longitudinal birth cohort. Cross‐lagged path analyses indicated a bidirectional relation between maternal emotion regulation difficulties and socio‐emotional problems over time, and a unidirectional relation between early child problems and later parenting stress. The findings illustrate how parents and children can influence each other's emotional well‐being in toddlerhood.

## 1 INTRODUCTION

Parents, and mothers in particular, are thought to shape children's socio‐emotional development through the quality of their caregiving, especially in early childhood (e.g., Eisenberg et al., 1998). Sensitive and responsive parenting during this period is important for a child's socio‐emotional development, supporting the socialization of emotion (Eisenberg & Spinrad et al., 1998). Individual parental factors may potentially underlie these parenting practices, yet these remain understudied (Morelen et al., 2016). Two of such factors thought to influence parenting and child socio‐emotional outcomes are parents’ capacity for emotion regulation and parenting stress (e.g., Crnic, 2024; Hajal & Paley, 2020). Following the parenting stress process model (Crnic, 2024), risk factors such as emotion regulation difficulties increase the vulnerability for parenting stress, which in turn is bidirectionally related to child behavioral problems. Thus, examining these parental factors longitudinally across toddlerhood increases our understanding of their interplay in relation to child outcomes (Edvoll et al., 2023).

### 1.1 Emotion regulation and socialization

The ability for emotion regulation, defined as the internal and external processes involved in initiating, evaluating, and modifying the occurrence, intensity, and expression of emotions to accomplish one's goals (Thompson, 1994), is still developing in early childhood. Consequently, following the theory of emotion socialization (Eisenberg & Spinrad et al., 1998), infants and toddlers rely on their parents for externally regulating their emotional reactions and behavior (e.g., by calming them in challenging situations). At the same time, toddlers experience increased autonomy and desire greater independence, resulting in more intrinsic regulation of emotions (e.g., using more intentional ways to self‐regulate) (Edvoll et al., 2023; Eisenberg & Spinrad, 2004). Thus, as toddlers are developing the foundations for intrinsic emotion regulation while still requiring external regulation, it follows that parents play a key role in shaping their emotional development during this period. The model of emotion socialization of Eisenberg and Spinrad (1998) proposed that parents foster children's emotion regulation abilities through their own expressiveness, their reactions to children's emotions, and through discussion of emotions (Eisenberg et al., 1998). Yet, for parents to facilitate their children's self‐regulatory skills, they must have sufficient emotional understanding and the ability to effectively emotions regulation themselves (Bariola et al., 2011; Hajal & Paley, 2020).

**KEY FINDINGS**

- Maternal emotion regulation difficulties and child socio‐emotional problems are positively, bidirectionally related throughout toddlerhood.
- Early child problems seem to affect later parenting stress within toddlerhood, while early parenting stress was not linked to child socio‐emotional problems at later timepoints.

**Statement of Relevance**

This study addresses the longitudinal effects of key parental risk factors on toddler mental health (i.e., socio‐emotional problems). The findings contribute to the field of infant mental health by examining the distinct contributions of parental emotion dysregulation and parenting stress, giving valuable insights into how mothers’ and toddlers’ well‐being impact each other throughout toddlerhood.

### 1.2 Parental emotion regulation difficulties

A capacity for effective emotion regulation implies emotional awareness, acceptance of emotions, and a certain degree of emotional control, ensuring that efforts to manage emotions align with current goals and contextual demands (Gratz & Roemer, 2004). Difficulties in emotion regulation indicate the absence of any or all of the above abilities and are associated with markers of maladjustment, underpinning numerous psychological disorders in adults (Joormann & Siemer, 2014; Tombini et al., 2020). As it is believed that parental emotion regulation is a crucial contributor to emotion parenting behaviors (Hajal & Paley, 2020), parental difficulties in emotion regulation may hinder the parent from reacting appropriately in stressful situations when their child requires their regulatory support. This has been shown in a recent study in which parents who reported more emotion regulation difficulties tended to have toddlers (2–3 years old) who showed poorer emotion regulation (Edvoll et al., 2023). This relation was not found in younger toddlers. Likewise, in older children, parents’ emotion regulation difficulties have been found to contribute to children's self‐regulation difficulties and behavioral problems (Zimmer‐Gembeck et al., 2022). Parenting practices are likely to have a mediating role in this relation (Palmer et al., 2020). Research indicated that fewer parental emotion regulation difficulties were associated with more positive parenting behaviors, which in turn were related to better emotion regulation, lower negativity, and fewer internalizing symptoms in children (Yan et al., 2021; Zimmer‐Gembeck et al., 2022). In contrast, maternal emotion dysregulation is associated with unsupportive parenting practices and with child emotion dysregulation (Morelen et al., 2016). However, most studies that investigated this relation included preschool or primary school‐aged children, while research in toddlers is lacking (Crespo et al., 2017; Morelen et al., 2016).

### 1.3 Parenting stress

Parenting stress has been defined as the psychological strain and feelings of overwhelm experienced by parents in response to stressors that can arise from the challenges and demands of parenting (Abidin, 1995). The model of Abidin attempts to identify the major components of parenting stress, focusing on proximal variables that are related to the demands of parenting, such as child characteristics, parents’ sense of competence, and social support. It proposes that parenting stress influences child functioning through its effect on parenting. A more recent model, the parenting stress process model of Crnic (2024), builds upon the core process outlined by Abidin, emphasizing the systemic nature of parenting stress. The model focuses on the transactional and bidirectional pathways between parenting stress, parent behavior, child behavior problems, and parent distress, and the moderators that influence each of these relations. In addition, Crnic's model includes the role of parental risk factors, such as emotion regulation difficulties, that may influence the degree of perceived parenting stress. Parents with stronger emotion regulation capacities have been shown to have better emotional coping abilities, making them more able to effectively deal with stressors (Deater‐Deckard et al., 2016). In contrast, parents’ emotion regulation difficulties may limit the emotional resources needed to deal with parenting stress, which has been shown to lead to higher levels of perceived stress and, in turn, less adaptive parenting practices and child behavioral problems (Crnic, 2024; Hu et al., 2019; Priego‐Ojeda & Rusu, 2023).

### 1.4 Parenting stress and child outcomes

While emotion regulation difficulties have been positively related to parenting stress, elevated levels of stress have, in turn, been associated with negative child socio‐emotional outcomes (e.g., Stone et al., 2016). Higher parenting stress in mothers has been found to contribute to fewer social skills and more internalizing and externalizing problems in their daughters (Carapito et al., 2020). In addition, higher parenting stress during infancy has been associated with later mental health problems and worse socioemotional and language development in children (Hattangadi et al., 2020; Troller‐Renfree et al., 2022). Importantly, parenting stress and child socio‐emotional outcomes, including behavioral problems, are thought to have a bidirectional relation (Crnic, 2024). Earlier findings revealed that earlier parenting stress predicted children's externalizing and internalizing problem behaviors at later ages, while earlier externalizing and internalizing behaviors were associated with later parenting stress (Jiang et al., 2023; Mackler et al., 2015; Neece et al., 2012).

### 1.5 Current study

There is a burgeoning literature pointing to the important relation between certain parental risk factors, such as emotion regulation difficulties and parenting stress, and child socio‐emotional outcomes. The conceptual process model of Crnic (2024) points to the relation between parental risk factors, such as emotion regulation and parenting stress, which in turn bidirectionally influence child adjustment. Nevertheless, a paucity of studies has investigated the interplay of these parental factors in the bidirectional relation to child socio‐emotional outcomes during toddlerhood. Thus, there is a need for longitudinal evidence investigating pathways between parental factors and child socio‐emotional outcomes within the first years postpartum (Morelen et al., 2016; Yan et al., 2021). It is important to investigate these relations, as they give a better understanding of when these parental risk factors may first impact socio‐emotional outcomes in children (Edvoll et al., 2023). Thus, this study aimed to investigate the longitudinal associations of parenting stress and maternal emotion regulation difficulties with child socio‐emotional problems within toddlerhood. To achieve this aim, we examined (a) whether there is support for a bidirectional relation between maternal risk factors and child socio‐emotional problems over time, within the first 3 years postpartum, and (b) whether maternal risk factors are interconnected, such that there is an indirect effect of emotion regulation difficulties via parenting stress on socio‐emotional problems. We hypothesized that maternal risk factors and child socio‐emotional problems are reciprocally and positively related within the first 3 years postpartum. Second, it was expected that parenting stress mediates the relation between emotion regulation difficulties on child socio‐emotional problems, as previous research has found that emotion regulation is related to parenting stress, which is in turn associated with child outcomes (Deater‐Deckard et al., 2016; Stone et al., 2016).

## 2 METHOD

The protocol for this study has been preregistered on OSF prior to data analyses and can be accessed at https://osf.io/68mgx/?view_only=08c464b5bd61431ea2a680a88d286cc1.

### 2.1 Participants

**FIGURE 1:**
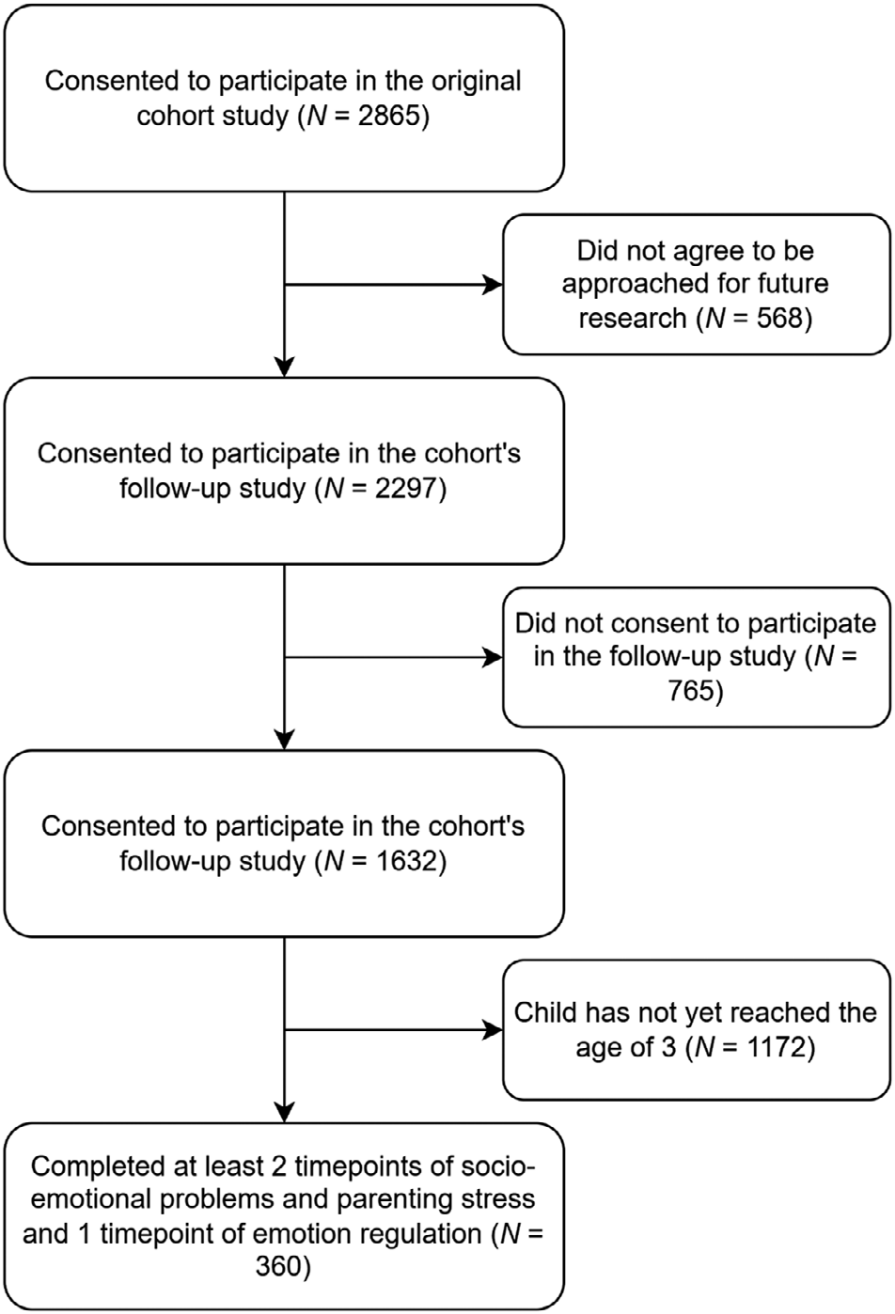
Enrollment of participants. Among participants in the follow‐up study, the only demographic difference between those included and not included in the current study was ethnicity (*p* = .015), with a higher proportion of Dutch‐origin mothers in the current sample.

The current study included 360 mothers. This is a subsample of mothers participating in the follow‐up of the Brabant Study, a large prospective perinatal cohort study examining obstetric outcomes from a biopsychosocial perspective from 12 weeks of pregnancy up until 5 years postpartum (Meems et al., 2020). Recruitment took place from 2018 to 2022. Mothers from the Southern regions of the Netherlands who were 18 years or older and had a sufficient understanding of the Dutch or English language were eligible for participation in the Brabant Study and received information about the study from their midwife at their first antenatal visit. Exclusion criteria were diabetes type I, rheumatoid arthritis, multiple pregnancy, known endocrine disorder before pregnancy (other than thyroid function problems), severe psychiatric disease, HIV, drug or alcohol addiction problems, or any other disease resulting in treatment with drugs that are potentially detrimental to the fetus. Starting at 6 months after childbirth, the participating mothers who agreed to be approached for future research were invited for the follow‐up study. The follow‐up study assesses maternal and child outcomes from 6 months to 5 years postpartum. The current study used assessments up to 3 years postpartum. In total, 2297 mothers were invited to participate in the follow‐up study, of which 1532 mothers (66.7%) gave their consent for participation. Mothers who completed at least two timepoints of socio‐emotional problems and parenting stress and one timepoint of emotion regulation difficulties within the first 3 years postpartum were eligible to participate in the current study. This resulted in a final sample of 360 mothers (*M*_age_ = 31.4 (SD = 3.4), 90.7 % Dutch, 81.4% completed university), who participated in the follow‐up from 2021 to May 2024 (see Figure 1 for an overview of enrollment). The original cohort study was approved by the medical‐ethical review board of the Máxima Medisch Centrum in Veldhoven (protocol number NL64091.015.17). The follow‐up study was approved by the Ethics Review Board of Tilburg University (protocol number RP41). All participants gave written informed consent.

### 2.2 Procedure

In the current study, mothers were invited to complete online questionnaires during the period spanning from 6 months to 3 years postpartum, via e‐mail using Qualtrics (www.qualtrics.com) every 6 months. Mothers could choose to fill in the questionnaires in either Dutch or English. In total, 84.2% of the mothers filled in the Dutch questionnaires. Emotion regulation difficulties were measured at 6 months and 1.5 years postpartum. Parenting stress and socio‐emotional problems were assessed at 1 year, 2 years, and 3 years postpartum.

### 2.3 Measures

#### 2.3.1 Emotion regulation difficulties

Maternal emotion regulation difficulties were measured with the short form of the Difficulties in Emotion Regulation Scale (DERS; Gratz & Roemer, 2004). This short form, the DERS‐16 (Bjureberg et al., 2016), has 16 items, which are rated on a 5‐point Likert scale from 1 = almost never to 5 = almost always. The items are divided into five subscales; (1) limited access to emotion regulation strategies (“when I am upset, I believe that there is nothing I can do to make myself feel better”), (2) difficulties controlling impulses (“when I am upset, I become out of control”), (3) lack of emotional clarity (“I am confused about how I feel”), (4) nonacceptance of emotional responses (“when I am upset, I feel like I am weak”) and (5) difficulties engaging in goal‐directed behaviors (“when I am upset, I have difficulty focusing on other things”). The current study uses total scores on the DERS‐16. Total scores ranged from 16 to 80, with higher scores indicating more parental emotion regulation difficulties. Psychometric properties of the DERS‐16 demonstrated good internal consistency (*α* = .92), convergent and discriminant validity, and test–retest reliability in a community sample in the United States (Bjureberg et al., 2016). The Dutch version of the DERS also showed good reliability (*α* > 72) in a sample of adolescents (Neumann et al., 2010). In the current study, reliability was excellent, with *α* = .94 and *α* = .95 for 6 months and 18 months postpartum, respectively.

#### 2.3.2 Parenting stress

To measure parenting stress, the Parenting Stress Questionnaire (PSQ) was completed by the participating mothers (Vermulst et al., 2012). The questionnaire can be completed by parents of children between 0 and 18 years of age. This scale consists of 34 items, rated on a 4‐point Likert scale from 1 = not true, 2 = somewhat true, 3 = quite true, 4 = very true. Three subscales of the PSQ were used in the current study; parent‐child relationship problems (six items; e.g., “I feel happy with my child”(reverse coded item)), parenting problems (perceived competence in parenting, seven items; e.g., “I can calm my child down when he/she gets angry”(reverse coded item)) and parental role restriction (six items; e.g., “raising my child leaves me with too little personal time”). Total scores combining the 19 items of the three subscales above range from 19 to 76, with higher scores indicating more parenting stress. In the current study, the range of values observed was 19–62. Both the Dutch and English versions of the PSQ were developed by Vermulst et al. (2012). Adequate reliability (McDonald's omega (*α*) = .90) and factorial validity of the PSQ *(χ* ^2^(517) = 2140.16; *p* < .001; Comparative Fit Index = .96 and Root Mean Squared Error of Approximation Index = .05) has been demonstrated in a sample of parents of children below 18 years old in the general Dutch population (Veerman et al., 2014). Likewise, the reliability of the three subscales in the current study has been found to be good, with *α* = .81, *α* = .82, and *α* = .82 for 1 year, 2 years, and 3 years postpartum, respectively.

#### 2.3.3 Socio‐emotional problems

The Brief Infant‐Toddler Social and Emotional Assessment (BITSEA) was used to assess child socio‐emotional problems (Briggs‐Cowan et al., 2002; Kruizinga et al., 2012). The questionnaire consists of two subscales: socio‐emotional competencies (11 items) and socio‐emotional problems (31 items). The former scale was excluded from analysis due to poor internal consistency and low test–retest correlations in previous studies (Briggs‐Cowan et al., 2004; Kruizinga et al., 2012) and a poor internal consistency in the current study (*α* = .598). The items (e.g., “often gets very upset”) are rated on a 3‐point Likert scale with answer options ranging from 0 = not true/rarely to 2 = very true/often, or as “not applicable.” A maximum of five items scored as “not applicable” in the socio‐emotional problems subscale was allowed to calculate the subscale score (Briggs‐Gowan & Carter, 2004). Total scores on this subscale ranged between 0 and 62, with higher scores indicating more socio‐emotional problems. The cut‐off scores for the at‐risk range of problems are 13–14 for girls and 14–15 for boys, depending on age. Psychometric properties of the BITSEA socio‐emotional problems subscale have been shown to be good, with adequate reliability and validity in samples of Dutch and American parents of 1–3‐year‐old children (*α* > .76) (Briggs‐Cowan et al., 2004; Kruizinga et al., 2012). Furthermore, these studies have shown that the BITSEA demonstrates excellent test–retest reliability and captures socio‐emotional difficulties that are relatively enduring, for some children, rather than transient within this age range. In the current study, the reliability of this subscale was found to be acceptable, with *α* = .74, *α* = .66, and *α* = .73 for 1 year, 2 years, and 3 years postpartum, respectively.

### 2.4 Statistical analysis

Preliminary analyses were performed in the Statistical Package for the Social Sciences (IBM SPSS version 28.0). First, demographic characteristics were calculated, and a missing value analysis was conducted. Independent samples *t*‐tests and Chi‐square tests of independence were conducted to check for differences between the complete cases and cases with missing values in the main variables of interest. Missing data were imputed using regression imputation in SPSS Amos, after which descriptive statistics were calculated. Child sex and maternal age were included as covariates in the analyses. Other demographics, including parental education level, marital status, and ethnicity, were not included as covariates due to minimal variability in the sample.

To test the main hypotheses, cross‐lagged path analyses were conducted in SPSS Amos Version 29.0. Cross‐lagged path analysis allows for simultaneous examination of the bidirectional pathways between parental factors and child outcomes over time. The path model included cross‐lagged paths linking each variable at each timepoint to subsequent timepoints of the other variables. In addition, auto‐regressive paths were estimated between the timepoints of each variable (see Figure S1 for the complete model). A Chi Square difference test was conducted to test whether the path from early child outcomes to later parental factors was significantly different from the path from early parental factors to later child outcomes at each cross‐lagged time point. Maximum Likelihood with bootstrapping (1000 bootstrap samples) and bias‐corrected confidence intervals were used, enabling the estimation of parameters and calculation of indirect effects. The criterion for statistical significance was set at *p* < .05. To determine good model fit, the following fit indices and cut‐off criteria were used (Hu & Bentler, 1999); (1) the Chi‐square test (χ2), where nonsignificant *p*‐values are expected with a CMIN/DF < 3 indicating an acceptable fit; (2) the Standardized Root Mean Square Residual (SRMR), where values below .08 indicate good fit; (3) the Tucker‐Lewis Index (TLI), where values above .95 are considered acceptable; (4) the Comparative Fit Index (CFI) where values above .95 are considered acceptable and (5) the Root Mean Squared Error of Approximation Index (RMSEA), with values up to .06 representing reasonable fit.

To examine the first hypothesis on bidirectional associations between maternal risk factors and child socio‐emotional problems over time, standardized path coefficients were calculated. Likelihood ratio estimates were used to examine potential differences in predictive value between parenting stress and emotion regulation difficulties. To examine the hypothesis on the mediating effect of parenting stress, the indirect effect from emotion regulation difficulties to child socio‐emotional problems via parenting stress was tested for significance using bootstrapping and bias‐corrected confidence intervals.

## 3 RESULTS

### 3.1 Preliminary analyses

Demographic characteristics of the sample can be found in Table 1. Mothers were on average 31 years old (*M* = 31.4, SD = 3.4, range = 23–43) and were mostly highly educated (81.4% completed university).

**TABLE 1:** Demographic characteristics.

	*N*	%	*M*	SD
Age mother (*N* = 355)			31.4	3.4
Sex child (*N *= 346): female	178	51.4		
Firstborn child (*N *= 358): yes	199	55.3		
Maternal education (*N* = 355)				
High school or lower	6	1.7		
Vocational education	60	16.9		
University	289	81.4		
Marital status (*N* = 330)				
Married/living together	326	98.8		
In a relationship without living together	4	1.2		
Socio‐cultural identity (*N* = 355)				
Dutch	322	90.7		
Other	33	9.3		

*Note*: *A*ll variables were measured at 12 weeks of pregnancy, except for marital status, which was assessed at 6 months postpartum, and child sex at 8 weeks postpartum.

Descriptives of the variables of interest from 6 months to 3 years postpartum are reported in Table 2. Missing value rates at 1 year, 2 years, and 3 years postpartum were as follows: 1.9%, 36.7%, and 37.2% for child socio‐emotional problems, and 1.9%, 10.8%, and 37.5% for parenting stress. For maternal emotion regulation difficulties, missing value rates were 9.4% and 7.8% for 6 months and 1.5 years postpartum, respectively. For the covariates, missing value rates were 1.4% for maternal age and 3.9% for child sex. A Little's Missing Completely at Random (MCAR) test was performed (*χ* ^2 ^= 157.06 (173), *p* = .802), indicating that the data were missing at random. Mothers who did not complete the parenting stress questionnaire (37.5%) or socio‐emotional problems questionnaire at 3 years postpartum (37.2%) did not differ on any of the main variables of interest or demographics (i.e., education level, maternal age, child sex) from mothers who did fill in this timepoint.

**TABLE 2:** Means and standard deviations of parenting stress, maternal emotion regulation difficulties, and child socio‐emotional problems at each time point.

	6mPP	1yPP	1.5yPP	2yPP	3yPP
Parenting stress					
*M* (SD)		29.7 (6.8)		29.4 (6.8)	30.2 (6.1)
Emotion regulation					
*M* (SD)	25.9 (10.2)		25.2 (10.3)		
Child problems^a^					
*M* (SD)		7.4 (4.3)		7.3 (4.1)	7.5 (4.1)

Abbreviations: 6mPP, 6 months postpartum; 1yPP, 1 year postpartum; 1.5yPP, 1.5 years postpartum; 2yPP, 2 years postpartum; 3yPP, 3 years postpartum.

^a^ Across time points, 6.9%– 9.2 % of the children had a total score above the cut‐off for the at‐risk range of problems, which was similar to the percentages found in a Finnish sample (Haapsamo et al., 2009).

### 3.2 Main analysis

#### 3.2.1 Model fit and path estimates

The model, including the maternal emotion regulation difficulties, parenting stress, child outcomes, child sex, and maternal age, showed good model fit according to all fit indices (*χ*2 (10) = 15.67; *p* = .110; CMIN/DF = 1.57; CFI = .996; SRMR = .02; TLI = .98; RMSEA = .04).

**FIGURE 2:**
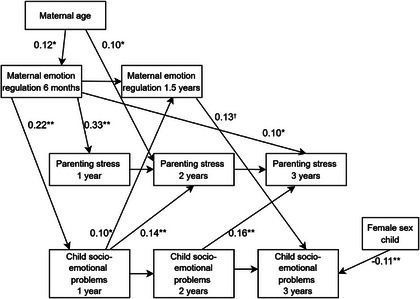
Cross Cross‐lagged path model including the standardized coefficients of the significant paths. *p < .05; **p < .01. Error terms connected to each variable are omitted in the model above for presentation purposes.

Standardized coefficients of the significant paths within the model are presented in Figure 2 and Table S2. First, the paths from early maternal risk factors to child socio‐emotional problems at later timepoints are assessed. Paths from maternal emotion regulation difficulties at 6 months to child socio‐emotional problems were statistically significant at 1 year postpartum (*β *= .22, CI [.13; .30], *p* = .003), yet not at 2 years postpartum (*β* = −.02, CI [−.12; .06], *p* = .627), nor at 3 years postpartum (*β* = .01, CI [−.09; .13, *p* = .825). Paths from maternal emotion regulation difficulties at 1.5 years to child socio‐emotional problems were not statistically significant at 2 years postpartum (*β* = .08, CI [−.001; .18], *p* = .102), yet marginally significant at 3 years postpartum (*β* = .13, CI [.02; .24], *p* = .051). As for parenting stress, none of the paths to child socio‐emotional problems were statistically significant. Thus, as for early maternal risk factors to later socio‐emotional problems, only emotion regulation difficulties at 6 months and 1.5 years postpartum were significantly positively associated with child problems at 1 year and 3 years postpartum, respectively.

As for the paths from early child socio‐emotional problems to maternal risk factors at later timepoints, findings indicated overall significant relations. The path from child socio‐emotional problems at 1 year to maternal emotion regulation difficulties at 1.5 years was statistically significant (*β* = .10, CI [.04; .17], *p* = .010). Likewise, the paths from socio‐emotional problems at 1 year and 2 years to parenting stress at 2 years and 3 years postpartum showed statistical significance as well; *β* = .14, CI [.07; .23], *p* = .006, and *β* = .16, CI [.10; .24], *p* = .001, respectively. Thus, a higher degree of socio‐emotional problems in children was associated with more maternal emotion regulation difficulties and higher levels of parenting stress at later timepoints.

For all variables, earlier timepoints were positively related to scores of later timepoints. The path from emotion regulation difficulties at 6 months to 1.5 years postpartum was statistically significant (*β* = .69, CI [.60; .75], *p* = .004). Likewise, the paths from parenting stress at 1 year to 2 years postpartum, and from 2 years to 3 years postpartum were statistically significant as well (Respectively: *β* = .51, CI [.40; .60], *p* = .002 and *β* = .35, CI [.25; .44], *p* = .002). Lastly, the paths from child socio‐emotional problems at 1 year to 2 years postpartum (*β* = .67, CI [.60; .73], *p* = .003) and from 2 years to 3 years (*β* = .56, CI [.46; .65], *p* = .002) showed statistical significance.

Furthermore, the Chi‐square difference test was used to test whether the paths from early child socio‐emotional problems to later parental factors were significantly different from the paths in the opposite direction (i.e., from early parental factors to later child problems). Results showed that the association between child socio‐emotional problems at 1 year postpartum and parenting stress at 2 years was significantly stronger (Δχ^2^(1) = 8.53, *p* < .001) than the reverse direction (i.e., from parenting stress at 1 year to child problems at 2 years). Similarly, the path from early child socio‐emotional problems (at 1 year) to later parental emotion regulation difficulties (at 1.5 years) differed significantly from the paths from early emotion regulation difficulties (at 1.5 years) to later child problems (at 2 years; Δ*χ* ^2^(1) = 5.33, *p* < .001 and at 3 years; Δ*χ* ^2^(1) = 4.43, *p* = .04). Thus, early socio‐emotional problems seem to be more strongly related to later parenting stress and emotion regulation difficulties compared to the reverse association.

#### 3.2.2 Covariates

Child sex was only associated with child socio‐emotional problems at 3 years (*β* = −.11, CI [−.18; −.05], *p* = .004), indicating that girls scored significantly lower compared to boys. Maternal age was significantly associated with maternal emotion regulation difficulties at 6 months postpartum (*β* = .12, CI [.03; .20], *p* = .019) and parenting stress at 2 years (*β* = .10, CI [.04; .15], *p* = .013). Higher maternal age was related to more emotion regulation difficulties and higher levels of parenting stress at these timepoints.

#### 3.2.3 Indirect effects

Lastly, the mediating role of parenting stress in the relation between emotion regulation difficulties and child socio‐emotional problems was tested (see Figure 2). First, maternal emotion regulation difficulties at 6 months postpartum were significantly positively related to parenting stress at 1 year (*β* = .33, CI [.26; .39], *p* = .003) and at 3 years (*β* = .10, CI [.03; .17], *p* = .019). Maternal emotion regulation difficulties at 1.5 years postpartum were marginally significantly related to parenting stress at 2 years (*β* = .09, CI [.01; .17], *p* = .050). Yet, the indirect paths from emotion regulation difficulties at 6 months and 1.5 years postpartum, via parenting stress at 1 year and 2 years, to child outcomes at 2 years and 3 years, respectively, were not statistically significant.

## 4 DISCUSSION

The current study examined the longitudinal associations between maternal emotion regulation difficulties, parenting stress, and child socio‐emotional problems in toddlerhood. Cross‐lagged path analyses indicated that maternal emotion regulation difficulties were related to more child socio‐emotional problems over time, and vice versa. Early child problems were associated with later parenting stress in mothers. Furthermore, more emotion regulation difficulties were related to higher parenting stress at later timepoints.

In line with the first hypothesis, a bidirectional relation was observed for maternal emotion regulation difficulties; early child socio‐emotional problems were linked to increased maternal emotion regulation difficulties at later timepoints, and vice versa. The association between maternal emotion regulation difficulties and child socio‐emotional problems has been found in previous research as well (Zimmer‐Gimbeck et al., 2020). As toddlers rely on their parents for externally regulating their emotions, this finding shows that parents must have sufficient ability for effective emotion regulation themselves to facilitate their toddlers’ self‐regulatory skills (Bariola et al., 2011). When parents struggle with emotion regulation, it can impede their ability to engage in supportive parenting during stressful situations when their child needs their regulatory support (Morelen et al., 2016). Consequently, children may experience more difficulties in emotion regulation, which contributes to the development of socio‐emotional problems. The current results revealed a significant relation between maternal emotion regulation difficulties and socio‐emotional problems at 1 year postpartum, marginally at 3 years postpartum, but not at 2 years. Conversely, previous research has found an effect of parental emotion regulation difficulties on child emotion regulation in older toddlers (ages 2–3), yet not in children under the age of 2 (Edvoll et al., 2023). One potential reason for this difference is that the current study used a different outcome measure, focusing on socio‐emotional problems rather than observed emotion regulation. Parental emotion regulation difficulties might be related to both younger (age 1) and older (age 3) toddlers’ observable socio‐emotional problems but might have a delayed relation with their intrinsic emotion regulation skills, as these skills are still developing. Furthermore, the lack of a significant association between early emotion regulation difficulties and child problems at 2 years in the current study might be attributed to relatively low reliability of the socio‐emotional problems assessment tool (BITSEA) at this timepoint (*α* = .66).

Moreover, the current study provides evidence for a relation between early child problems and later emotion regulation difficulties in mothers. This finding is particularly notable, as the association between early child behavior and later parental emotion regulation is much less studied. The results suggest that emotional contagion may occur due to the extensive time mothers and toddlers spend together (Butler et al., 2015; Tan & Smith, 2022). Consequently, a child's problems can directly affect the mother's emotional state and emotional regulation ability. Toddlers with socio‐emotional problems may exhibit challenging behaviors that increase stress and caregiving demands, impairing a mother's ability to regulate her own emotions over time. Similarly, it has been suggested that children's negative affectivity (i.e., temperamental frustration, sadness, and fear) and maternal negative emotion expressivity are bidirectionally related from toddlerhood to school‐age (Tan & Smith, 2022). These findings highlight the need for early intervention programs that address not only children's socio‐emotional problems but also provide support for parents, preventing the development of maternal emotion regulation difficulties (Kohlhoff et al., 2020). Findings thus imply that a holistic approach that includes parental emotional resilience is essential, recognizing how closely child and parent emotional well‐being are linked.

Regarding parenting stress, the current results do not support a bidirectional relation between parenting stress and child socio‐emotional problems, as proposed by the process model of Crnic (2024). Although early child problems were linked to parenting stress at later timepoints, the reverse was not observed. This may be due to the age of the children included in the current study. Previous research indicating a bidirectional relation has primarily involved children older than 3 years (Jiang et al., 2023; Mackler et al., 2015; Neece et al., 2012). Consistent with our results, Van Dijk et al. (2022) and Kochanova et al. (2022) reported bidirectional relations between parenting stress and child behavioral problems in older children and adolescents, yet not over time in early childhood (age 1–5). These results suggest that young children may be less aware of and impacted by parenting stress. However, some studies do find an association between higher parenting stress during infancy and early toddlerhood (under 18 months) and more socio‐emotional problems in 3‐year‐old children (Hattangadi et al., 2020; Tharner et al., 2012). Tharner et al. (2012) found this association only in insecurely attached children. Differences in covariates assessed may explain the discrepancies in findings. In the study of Hattangadi et al. (2020), for instance, household income seemed to be predictive and may account for the association between parenting stress and child socio‐emotional problems. As in the current study, the sample of Hattangadi et al. (2020) consisted predominantly of well‐educated mothers. Another explanation might be that parenting stress is only associated with child adjustment among mothers with poorer emotion regulation skills. When mothers are able to regulate their parenting stress effectively, it may not negatively impact toddlers’ socio‐emotional development. Although findings in mothers are limited, a similar moderating role of maladaptive emotion regulation has been observed in the relationship between life stressors and psychological outcomes in adolescents (Klosowska et al., 2020).

Furthermore, findings did not support the second hypothesis, showing no mediating role of parenting stress in the relation between maternal emotion regulation and socio‐emotional problems. Nevertheless, emotion regulation difficulties were significantly associated with later parenting stress. This finding is in line with the process model of Crnic (2024) that parental emotion regulation difficulties are a risk factor for perceived parenting stress by limiting the emotional resources needed to deal with distress. It was hypothesized that, in turn, exposure to a stressed parent may have a dysregulating effect on the child's stress response, which is associated with more socio‐emotional problems (Huth‐Bocks & Hughes, 2008). Yet, the results suggest that this association between early parenting stress and later child socio‐emotional problems is not consistently apparent in toddlerhood. This leaves room for future research to investigate the contexts in which this relation may emerge in young children, such as insecure attachment (Tharner et al., 2012), low socioeconomic status (Hattangadi et al., 2020), or other familial risk factors.

Importantly, the present findings do show a unidirectional relation between early child socio‐emotional problems and later parenting stress. These results confirm Abidin's model (1995), showing that parenting stress increases when the parent views their child as being behaviorally more difficult. Children with more socio‐emotional problems often increase parenting demands, which, if not matched with available resources for meeting these demands (e.g., social support, knowledge), can lead to higher levels of parenting stress. The pattern found in the present study is consistent with findings in children aged 5–15, indicating that children's early behavioral problems predicted parenting stress at later timepoints, while the reverse effect was not observed (Jiang et al., 2023). Therefore, the results suggest that while parenting stress does not exacerbate children's behavioral problems at this age, children's problems are related to increased parenting stress in mothers during toddlerhood.

Concerning the covariates, child sex was associated with socio‐emotional problems at 3 years postpartum, with girls scoring significantly lower than boys. This aligns with previous research indicating that boys are reported to have more socio‐emotional problems than girls at this age (Eurenius et al., 2018; Kruizinga et al., 2012). An important consideration is that this observed sex difference may be partly explained by boys being more likely to externalize their socio‐emotional difficulties, making these behaviors more observable and, consequently, more likely to be reported by parents (Eurenius et al., 2018). Adequately measuring socio‐emotional difficulties may therefore be difficult within this age range, as assessments mainly rely on parent‐report. Nevertheless, in the study of Briggs‐Cowan et al. (2004), the observational assessment of child socio‐emotional problems correlated with the parent‐reported scores on the BITSEA problem scale. This highlights the importance of incorporating observational methods, such as emotion‐eliciting or frustrating lab tasks (Calkins & Johnson, 1998). Furthermore, higher maternal age was related to increased emotion regulation difficulties at 6 months postpartum and higher levels of parenting stress at 2 years postpartum. Previous research has not often included parental age in the main analyses. Further research is needed to examine the interaction between maternal age and postpartum maternal psychological factors.

It is important to also acknowledge some limitations of the current study. A first limitation pertains to the chosen statistical model, as estimated path coefficients in a cross‐lagged path model may reflect a mixture of within‐ and between‐subjects effects. Future studies are encouraged to test alternative models, such as the autoregressive latent trajectory (ALT) model with structured residuals or the random intercept cross‐lagged panel model (RI‐CLPM) (Curran et al., 2014). Second, we did not include variables relating to parenting practices, despite research indicating that these practices likely mediate the relation between parental risk factors and child outcomes (Carapito et al., 2020; Morelen et al., 2016; Palmer et al., 2020). For future studies investigating the relation between maternal risk factors and child socio‐emotional problems, it would be beneficial to include some factors related to parenting practices or family dynamics (e.g., parental self‐efficacy, partner involvement, household income, adverse life events) or an emotion regulation measure specific to the parenting context (e.g., the Parent Emotion Regulation Scale; Pereira et al., 2017). Furthermore, the current study only included socio‐emotional problems and no other aspects of socio‐emotional development, such as social skills. Since different patterns of relations with parental risk factors could emerge, future studies should consider exploring these additional dimensions of child development. Third, participating mothers were generally married, highly educated, and few had an ethnic minority background, which may limit the generalizability of the findings. This context may also explain the findings regarding parenting stress. In mothers from an ethnic minority or single parents, levels of parenting stress might be higher (Nam et al., 2015; Williford et al., 2007), which could reveal a relation between parenting stress and child outcomes that was not observed in this sample. Lastly, mothers self‐reported their emotion regulation difficulties, parenting stress, and their children's socio‐emotional problems, which might potentially bias the results. For instance, parenting stress can affect how parents perceive their child's behavior, potentially leading those who experience early parenting stress to view their child's behavior as more problematic. Due to shared method variance, this may have led to inflated associations among variables. In addition, parental self‐reports may have been affected by the COVID‐19 pandemic, as the current data were partly collected during this period. Previous research suggests that the pandemic may have increased parenting stress, which may in turn be related to how parents report child socio‐emotional problems (Dillmann et al., 2022; Wiley et al., 2023). To address these self‐report biases, future research could include assessments by additional caregivers or teachers and include more observational data collection. A final limitation to consider here is that attrition at 3 years postpartum was relatively high compared to earlier timepoints, likely due to general participant attrition over time. Nevertheless, a strength of the present study is the longitudinal design with multiple assessments across toddlerhood. This enabled the analyses of bidirectional pathways and allowed the examination of both direct and indirect relations between parental risk factors and child socio‐emotional problems.

Taken together, the current study provides important insights into the reciprocal, longitudinal relations between maternal risk factors and child socio‐emotional problems in toddlerhood. The results indicate that socio‐emotional problems are significantly related to maternal risk factors (i.e., parenting stress and maternal emotion regulation difficulties) throughout toddlerhood, aligning with research in older children. On the other hand, toddlers seem to be less impacted by parenting stress, finding no relation between early parenting stress and later socio‐emotional problems, as found in older children. Maternal emotion regulation difficulties, however, seem to be specifically related to socio‐emotional problems in younger toddlers. This is likely due to the heightened emotional contagion at this age, as mothers and young toddlers spend most of their time in close interaction. These results, illustrating how mothers and toddlers influence each other's emotional well‐being, emphasize the importance of considering these dynamic interactions in early intervention and support programs. Nonetheless, the findings should be interpreted with caution, as the statistical model used (i.e., cross‐lagged path model) does not entangle within‐ and between‐person effects, underscoring the need for future research using an adjusted modelling approach.

## CONFLICT OF INTEREST STATEMENT

The authors declare no conflict of interest.

## Supporting information



Supporting Information

## Data Availability

Data and materials are available from the corresponding author upon reasonable request. The analytic code necessary to reproduce the analyses presented in this paper is not publicly accessible. The analyses presented here were preregistered. The preregistration is available at the following URL: https://osf.io/68mgx/view_only=08c464b5bd61431ea2a680a88d286cc1
