# Supplementary material for: A cross‐lagged panel model examining the longitudinal associations between maternal emotion regulation difficulties, parenting stress, and child socio‐emotional problems in toddlerhood
Source: Infant Ment Health J. 2025 Oct 6;47(1):e70051. doi: 10.1002/imhj.70051 (PMC12719906; doi:10.1002/imhj.70051)
Supplement: Supplementary file 1 — Supporting Information [file IMHJ-47-0-s001.docx]

Supplemental Materials Figure S1

*Cross-lagged panel model in SPSS Amos*

*
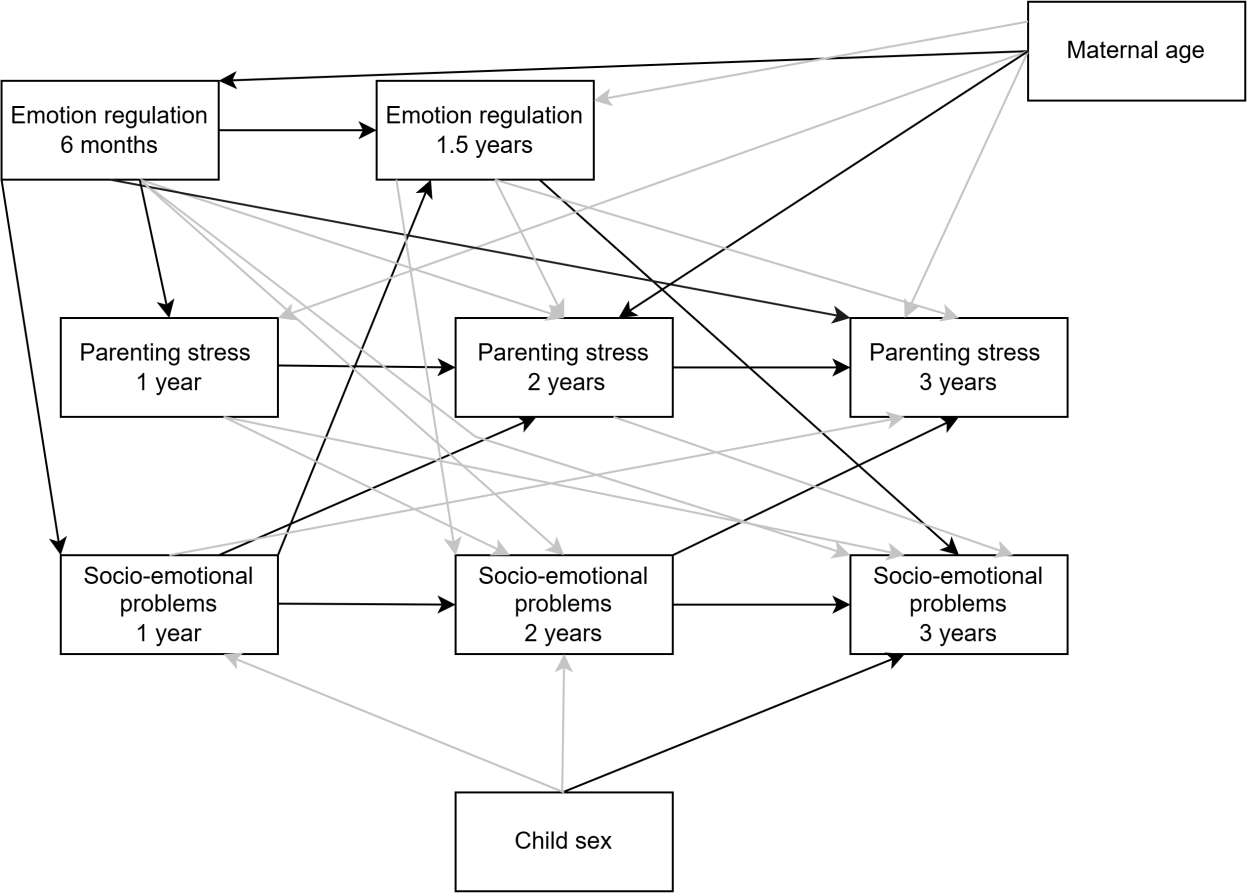
*

*Note.* Black arrows represent significant paths (*p* < .05), grey arrows represent insignificant paths. Error terms connected to each variable are omitted in the model above for presentation purposes.

Table S1

*Standardized coefficientsand standard errors for all paths*

|  | **Standardized regression weight** | **Standard error** |
| --- | --- | --- |
| Maternal age > DERS_6mPP | .12 | 0.05 |
| Maternal age > DERS_1y6mPP | -0.04 | 0.04 |
| Maternal age > OBVL_1yPP | 0.04 | 0.04 |
| Maternal age > OBVL_2yPP | 0.10 | 0.03 |
| Maternal age > OBVL_3yPP | -0.01 | 0.03 |
| Sex child > BITSEA_1yPP | 0.03 | 0.05 |
| Sex child > BITSEA_2yPP | -0.05 | 0.04 |
| Sex child > BITSEA_3yPP | -0.11 | 0.04 |
| DERS_6mPP > BITSEA_1yPP | 0.22 | 0.05 |
| DERS_6mPP > OBVL_1yPP | 0.49 | 0.05 |
| DERS_6mPP > DERS_1y6mPP | 0.69 | 0.04 |
| DERS_6mPP > BITSEA_2yPP | -0.02 | 0.05 |
| DERS_6mPP > BITSEA_3yPP | 0.01 | 0.07 |
| DERS_6mPP > OBVL_2yPP | 0.03 | 0.07 |
| DERS_6mPP > OBVL_3yPP | 0.16 | 0.07 |
| DERS_1y6mPP > OBVL_2yPP | 0.13 | 0.07 |
| DERS_1y6mPP > BITSEA_2yPP | 0.08 | 0.06 |
| DERS_1y6mPP > BITSEA_3yPP | 0.13 | 0.07 |
| DERS_1y6mPP > OBVL_3yPP | 0.10 | 0.07 |
| BITSEA_1yPP > DERS1y6mPP | 0.10 | 0.04 |
| BITSEA_1yPP > OBVL_2yPP | 0.14 | 0.05 |
| BITSEA_1yPP > BITSEA_2yPP | 0.67 | 0.04 |
| BITSEA_1yPP > BITSEA_3yPP | 0.09 | 0.06 |
| BITSEA_1yPP > OBVL_3yPP | -0.03 | 0.05 |
| BITSEA_2yPP > BITSEA_3yPP | 0.56 | 0.06 |
| BITSEA_2yPP > OBVL_3yPP | 0.16 | 0.05 |
| OBVL_1yPP > OBVL_2yPP | 0.51 | 0.06 |
| OBVL_1yPP > BITSEA_2yPP | -0.004 | 0.05 |
| OBVL_1yPP > BITSEA_3yPP | -0.01 | 0.06 |
| OBVL_1yPP > OBVL_3yPP | 0.26 | 0.06 |
| OBVL_2yPP > OBVL_3yPP | 0.35 | 0.06 |
| OBVL_2yPP > BITSEA_3yPP | -0.01 | 0.05 |
